# Preparation and Characterization of κ-Carrageenan Modified with Maleic Anhydride and Its Application in Films

**DOI:** 10.3390/md19090486

**Published:** 2021-08-26

**Authors:** Yuan Zhou, Fu-Quan Chen, Si Chen, Qiong Xiao, Hui-Fen Weng, Qiu-Ming Yang, An-Feng Xiao

**Affiliations:** 1 College of Food and Biological Engineering, Jimei University, Xiamen 361021, China; 201811832012@jmu.edu.cn (Y.Z.); fqchenhy0109@jmu.edu.cn (F.-Q.C.); 201912951025@jmu.edu.cn (S.C.); xiaoqiong129@jmu.edu.cn (Q.X.); 201572000057@jmu.edu.cn (H.-F.W.); yangqm@jmu.edu.cn (Q.-M.Y.); 2 National R&D Center for Red Alga Processing Technology, Xiamen 361021, China; 3 Fujian Provincial Engineering Technology Research Center of Marine Functional Food, Xiamen 361021, China; 4 Xiamen Key Laboratory of Marine Functional Food, Xiamen 361021, China

**Keywords:** κ-carrageenan, maleic anhydride, modification, physicochemical property, film

## Abstract

In this work, the physicochemical properties of maleic anhydride (MAH)-modified κ-carrageenan (κCar) (MC) were characterized and compared with those of native κ-carrageenan (NC). The Fourier transform infrared spectrum of MC exhibited that κCar was successfully modified. Thermogravimetric analysis indicated that the thermal stability of MC was decreased. When the degree of substitution was 0.032, MC exhibited a low gel strength (759 g/cm^2^), gelling temperature (33.3 °C), and dehydration rate (60.3%). Given the excellent film-forming ability of κCar, MC films were then prepared and were found to have better mechanical and barrier properties (UV and water) than NC films. With regard to optical properties, MC films could completely absorb UV light in the range of 200–236 nm. The water contact angle of MC films was higher than that of NC films. Moreover, the elongation at break increased from 26.9% to 163%. These physicochemical property changes imply that MC can be employed in polysaccharide-based films.

## 1. Introduction

Carrageenan is a naturally linear polysaccharide that consists of alternating 3-linked β-d-galactopyranose and 4-linked α-d-galactopyranose or 4-linked 3,6-anhydro-α-d-glactopyranose, forming the disaccharide repeating unit of carrageenan [1]. As a type of sulfated polysaccharide, carrageenan can be categorized into κ- (kappa), ι- (iota), and λ- (lambda) types according to the presence of the 3,6-anhydro-bridge on the 4-linked-galactose residue and the position and number of sulphate groups [2]. Given its gelling, thickening, emulsification, stability, and non-toxicity properties, κ-carrageenan (κCar) is widely used in food and commodity industries [3], such as to thicken ice cream and milk drinks or as an emulsifier and stabilizer in toothpaste, cosmetic creams, and shampoos.

However, the use of κCar processed by traditional methods is limited because of its disadvantages, such as high gel strength, easy shrinkage, and high melting temperature. For instance, the high gel strength of κCar reduces the fluff and taste of pastry and steamed bread and increases the solidification of shampoo and toothpaste [4]. In many cases, the water retention of κCar might be below the required value. A gel with high water retention has good water holding stability and soft gel properties, thus endowing the product with stable tissue morphology and soft elastic characteristics [5]. Sinthsamran et al. [6] found that the high water holding capacity of κCar could prevent free water precipitation in meat products for preservation at a certain period. Moreover, its low melting and gelling temperature allows carrageenan to be combined with thermosensitive substances and thus save energy. Hence, different modification methods have been developed to modulate the physicochemical characteristics of κCar and thus improve its quality or expand its applications. Savoji and Pourjavadi [7] investigated a novel superabsorbent hydrogel based on κCar and polyacrylonitrile. A super swelling hydrogel with a water absorption capacity of up to 3260 g/g (water/dry hydrogel powder) was obtained under optimized conditions. Papageorgiou et al. [8] studied novel isocyanate-modified carrageenan polymers as sorbent materials.

Maleic anhydride (MAH) is an important unsaturated organic acid anhydride with a strong oxidation property and can esterify hydroxyl compounds with high steric hindrance [9]. MAH-esterified starch, which was produced by using corn starch as the material and MAH as the esterifying agent via a dry method, could increase the length of the starch side chain and consequently enhance thermoplastic characteristics [10]. The modification of κCar with MAH has been reported [11,12]. However, reports on the modification of κCar with MAH only and focusing on its physicochemical properties, such as gelling and thermal properties, are almost limited (to the best of the authors’ knowledge). We hypothesize that MAH can replace one of the κCar hydroxyl groups and be grafted onto its structural unit. In consideration of the κCar gelation mechanism [13], introducing MAH groups to κCar molecules would form “kinks” and repulsive groups in molecular chains, reduce the binding force, and prevent the synthesis of double helix chains, thus weakening the gel strength of κCar [14]. Inserting a bulky MAH group may reduce the interaction of adjacent κCar chains and promote discontinuity in the hydrogen bonding. Incorporating bulky groups on the κCar chains may also increase the ability of gels to retain water, thereby enhancing the water holding capacity [15]. The side chain length and the molecular chain spacing of κCar will also increase [16], whereas the intermolecular force will decrease, thus reducing the gelling temperature. These studies indicate that MAH modification transforms κCar as a new feature hydrocolloid with low gel strength and gelling temperature but high water holding capacity.

As a biodegradable polysaccharide, κCar has excellent film-forming features [17,18]. However, similar to most polysaccharide-based films, plain κCar films have some limitations due to their inherent hydrophilic nature that negatively affects their mechanical and water barrier properties as compared with normal synthetic polymers [19]. Numerous physical and chemical techniques have been developed to obtain desirable film properties. Blending with polymers [20], reinforcing with nanomaterials [21], or layering with other polymer films [22] can remarkably enhance the film properties of carrageenans.

In this study, the effects of MAH on the characteristics of κCar films were evaluated to expand the application of κCar in polysaccharide-based films. MAH-modified κCar was successfully prepared and films based on the mixed MAH-modified κCar/glycerol were prepared via solution casting. The major factors affecting MAH modification and the physicochemical properties of MC were studied. The microstructure, optical properties, mechanical properties, and water affinity of these modified κCar films were also determined and compared with those of native κCar films. In conclusion, the chemical modification of κCar with MAH is an effective and promising pathway to endow κCar with new properties and consequently improve the features of κCar-based films.

## 2. Results and Discussion

### 2.1. Optimization of Reaction Conditions for MC Synthesis

MAH forms maleic acid and esters when dissolved in water and ethanol, respectively, thus creating a mixture in the reaction [23]. Previous studies mostly focused on the reaction of MAH with starch and rarely on the reaction of MAH with κCar. Zuo et al. [10] reported that MAH modification for starch occurs mainly in the amorphous region of the granule with the maximum degree of substitution (DS) of 0.3386. The availability of -OH groups at C-2, C-3, and C-6 (glucose unit, Mw 162) renders native starch reactive for substitution [24]. κCar consists of alternating (1→3)-β-d-galactose-4-sulfate and (1→4)-3,6-anhydro-α-d-galactose residues joined in a linear chain, in which the available -OH groups possess different reactivities for esterification [1]. Similar to starch, κCar has an extremely high water-absorbing capacity to react easily. However, κCar has no crystalline region, which means that the water-absorbing capacity of κCar is higher than that of starch. Therefore, κCar has less reactivity than starch, leading to a lower DS. Gel strength is also an important index for κCar. High gel strength is not always the most desirable quality for κCar. However, low strength bestows the product with a stable structure and soft elastic characteristics. κCar with low gel strength has potential uses in liquid food, spread food, soft-texture confectionery, fat replacers, and new industrial applications, and can also serve as a cryoprotectant to minimize the damage of freezing–thawing [25,26]. Thus, the effects of five factors on the DS and gel strength of MC were studied.

**Figure 1:**
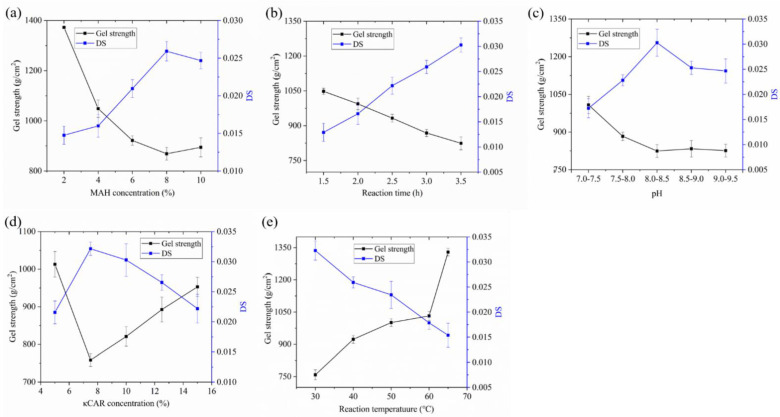
Effects of reaction conditions on modification. (**a**) Effect of MAH concentration on modification: reaction time 3.5 h, pH 8–8.5, κCar concentration 7.5%, and reaction temperature 30 °C; (**b**) Effect of reaction time on modification: MAH concentration 8%, pH 8–8.5, κCar concentration 7.5%, and reaction temperature 30 °C; (**c**) Effect of pH on modification: MAH concentration 8%, reaction time 3.5 h, κCar concentration 7.5%, and reaction temperature 30 °C; (**d**) Effect of κCar concentration on modification: MAH concentration 8%, reaction time 3.5 h, pH 8–8.5, and reaction temperature 30 °C; (**e**) Effect of reaction temperature on modification: MAH concentration 8%, reaction time 3.5 h, pH 8–8.5, and κCar concentration 7.5%. The reaction was carried out by changing one parameter in each case but keeping the other 4 parameters constant (pooled data from three experiments are presented as means ± standard deviation of the mean (n = 3)).

The effects of MAH concentration on DS and gel strength are illustrated in Figure 1a. Each κCar structural unit has three -OH groups and one sulphate group. MAH could replace one of the hydroxyl groups and be grafted onto the κCar structural unit [11]. The concentration of MAH possibly affected the diffusion of MAH into the κCar molecules. When the MAH concentration was increased from 2% to 8%, the available MAH groups around the κCar molecule increased, leading to an increase in DS. However, an overly high concentration of MAH was unfavorable for the substitution. In order to maintain the reaction pH constant, it was necessary to add vast quantities of alkali, which resulted in the swelling of κCar and difficulty to agitate the reaction system, thus reducing the DS. Moreover, gel strength was negatively correlated with DS. This result may be attributed to the introduction of substituents that broke and loosened up the hydrogen bonding between κCar molecules [27], which prevented the synthesis of double helix chains. Thus, 8% was selected as the optimal MAH concentration in the subsequent experiments.

According to Figure 1b, the DS of MC was steadily increased when the reaction time was prolonged from 1.5 h to 3.5 h. This result could be interpreted by the increased contact and collision between the MAH and κCar with the prolonged reaction time, which was similar to the report described previously [28]. In the present study, the gel strength was decreased with prolonged reaction time because the introduction of esterification groups to κCar molecules can reduce the interaction of adjacent κCar chains and promote discontinuity in the hydrogen bonding [14]. In addition, the disruption of hydrogen bonds also facilitates water penetration to the interior of the particles [29]. Thus, the κCar being in the lye for a long period of time might damage its structure and reduce its gel strength.

Tailoring the pH of the reaction system is important for the modification of κCar, and during modification, NaOH was used for keeping the pH constant. Figure 1c exhibits the effect of the pH of the reaction system on the modification of κCar. DS rapidly increased when the pH was increased from 7.0 to 8.5. This finding may be related to the fact that effective alkalinity could promote the nucleophilicity of the hydroxyl group and swell the κCar particles, resulting in higher substitution; while with a further increase in NaOH amount, the hydrolysis of MAH and MC was accelerated, resulting in a decrease in DS [16]. The gel strength was not increased with the decreasing DS, indicating that this parameter was highly affected by alkalinity. Therefore, the appropriate reaction pH was 8–8.5.

To determine the influence of κCar concentration, the experiment was performed, and the result is shown in Figure 1d. The DS reached the maximum when the κCar concentration was increased to 7.5% (*w*/*v*). A continuous increase in κCar concentration resulted in a decrease in DS. This result was due to the swelling of κCar, which was difficult to agitate in the reaction system. Ruan et al. [30] also found a similar result of octenyl succinic anhydride modified starch. Therefore, it was possible to obtain MC with a higher DS under the concentration of 7.5% κCar.

The effect of reaction temperature on the modification of κCar is illustrated in Figure 1e. With the increase in reaction temperature from 30 °C to 65 °C, the DS gradually decreased from 0.032 to 0.015. The reactant activity and reaction rate decreased with the increasing temperature because the esterification was exothermic. Thus, MC with a DS of 0.032 and gel strength of 759 g/cm^2^ was obtained when the reaction temperature was 30 °C.

### 2.2. Characterization of MC

#### 2.2.1. Fourier Transform Infrared (FT-IR) Analysis

**Figure 2:**
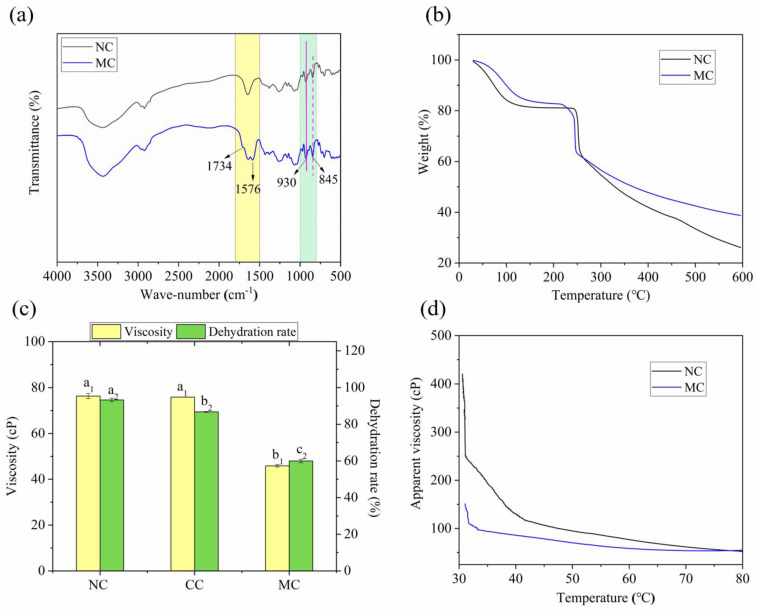
(**a**) FT-IR spectra; (**b**) TGA spectra of NC and MC; (**c**) viscosity and water-holding capacity of κCar samples; (**d**) viscosity–temperature curves of NC and MC; CC: control κCar. a_1_, b_1_ on the bar graph indicate significant differences in viscosity (*p* < 0.05) and a_2_, b_2,_ c_2_ indicate significant differences in dehydration rate (*p* < 0.05) in Figure 2c.

FT-IR was used to investigate the changes in κCar after modification, as shown in Figure 2a. The characteristic absorption peaks of κCar appearing at 845 and 930 cm^−1^ contributed to the C–H bending of substituted d-galactose-4-sulfate and the C–O bond of 3,6-anhydro-d-galactose (3,6-AG), respectively [12]. The extremely broad band at approximately 3440 cm^−1^ corresponded to the vibration of the hydroxyl groups (O–H). In addition, the band at 1250 cm^−1^ was characteristic of S=O of sulfate esters. Compared with NC, MC (DS = 0.032) exhibited new peaks at 1576 and 1734 cm^−1^. The peak at 1576 cm^−1^ corresponded to the asymmetric stretching vibration of the carboxylate (RCOO−), whereas the peak at 1734 cm^−1^ suggested the formation of ester carbonyl groups (C=O) between the κCar and MAH [31]. These two additional peaks of MC confirmed that κCar was successfully modified by MAH, which was in agreement with a previous study [11].

#### 2.2.2. Thermogravimetric Analysis (TGA) 

Figure 2b shows the TGA profiles of NC and MC. Weight loss in NC and MC occurred in three main temperature phases. NC and MC (DS = 0.032) partially lost weight in the temperature range of 30–160 °C as a result of free water reduction. Given the same moisture contents of NC and MC after drying, their water holding capacity could be defined by their water loss rate. The water loss rate of MC was lower than that of NC, probably indicating that MC had higher water holding capacity than NC. At the temperature range of 200–250 °C, NC and MC showed a sharp mass loss of 20%, suggesting the rapid decomposition of κCar at this stage. Moreover, the initial degradation temperature of the MC was lower than that of the NC, indicating that the introduction of the MAH group may negatively affect the thermal stability of κCar. A similar result was reported by Chen et al. [32], who found that the thermal stability of agar also declined after chemical modification. When the temperature was increased up to 600 °C, the decomposition then proceeded slowly. The higher remaining residue of MC than that of NC could be attributed to the introduction of MAH groups.

### 2.3. Physicochemical Properties 

#### 2.3.1. Viscosity and Water Holding Capacity

As shown in Figure 2c, the viscosity of MC (DS = 0.032) was 39.9% lower than that of NC, and this value was conducive to the preparation of MC film-forming solution. A similar pattern regarding viscosity was observed in starch esterified by octenyl succinic anhydride [33]. The decreased viscosity of MC could be due to the breakage of hydrogen bonds and the weakening of intermolecular forces of carrageenan after the introduction of MAH groups. In addition, the introduced branched chains may hinder the intertwining of the molecular chains of carrageenan, resulting in the reduced viscosity of MC. Furthermore, the partial hydrolysis of carrageenan chains under alkaline esterification may also decrease the viscosity [34].

Compared with that from NC, the water released from MC (DS = 0.032) was 33.0% lower after freezing–thawing, as shown in Figure 2c. Therefore, MC had better water holding capacity than NC, and this finding was consistent with the TGA results. Several factors may account for this phenomenon. On the one hand, the double helixes of carrageenan were reduced after MAH esterification, thus exposing many hydroxyl groups that formed hydrogen bonds with water and improved the water holding capacity. On the other hand, the branching structure of MC increased the side chain length and steric hindrance of the molecular chain and consequently the water holding capacity. The ability of MC to retain large amounts of water allows for stabilized suspensions in emulsions and provides great resistance to deformation; this feature has some potential applications in biomedicine, refrigeration, the food industry, and cosmetics [35]. Meindrawan et al. [36] reported that nanocomposite coatings based on κCar and ZnO nanoparticles could maintain the storage quality of mango by decreasing water permeability and increasing the water holding capacity. 

The apparent viscosity of MC (DS = 0.032) solution was basically lower than that of NC at the same temperature, as shown in Figure 2d. Initially, the apparent viscosity of the samples was basically stable from 70 °C to 80 °C. Thereafter, the increase in viscosity became apparent as the temperature was lowered. When the temperatures of NC and MC were further reduced to 40 °C and 33 °C, their apparent viscosities started to increase sharply with the decrease in temperature, indicating that the κCar molecules started to form gels. Therefore, the gelling temperature of MC was lower than that of NC.

#### 2.3.2. Other Properties

The physical properties of the κCar samples are illustrated in Table 1. It could be seen that the MAH modification significantly influenced the gel strength and the 3,6-AG content of κCar. As the DS increased from 0.015 to 0.032, both the gel strength and 3,6-AG of MC decreased. Among them, the decreased gel strength of MC may be attributed to the introduction of MAH groups and electrostatic repulsion from the carboxylate groups, thereby hindering the aggregation of helical conformation [32]. The reduced 3,6-AG content in MC was possibly caused by the degradation of κCar by MAH. In addition, the sulfate content of MC was not significantly changed compared to that of NC. 

Variations in transparency occur due to the penetration, refraction, and reflection of light at different intensities when irradiating different solutions. Transparency affects product quality, properties, and uses. Carrageenan with high transparency is widely used. Highly transparent carrageenan film for observational experiments can be prepared by mixing with glycerol. As shown in Table 1, the transparency of MC increased remarkably from 81.9% to 91.6% by 11.8% with the increase in DS. The results could be explained as follows: the introduction of MAH groups prevented the carrageenan molecules from binding to each other and the formation of the double helix structure, thus allowing light to penetrate easily [37].

The dissolution of carrageenan in water can be divided into two steps: first, carrageenan swells and partially hydrates, and the polymer chain is then broken and dissolved [13]. In the present research, the MC powder could be dissolved in water at 93.1 °C when the DS was 0.032. Esterification can weaken the internal hydrogen bond of carrageenan molecules and easily unite the polymerization chains [32]. Moreover, the degradation of carrageenan by MAH can destroy the partial molecular structure of carrageenan. Therefore, carrageenan could be dissolved at a low temperature, thus creating a favorable condition for the preparation of film-forming solution.

The gelling temperature of MC (DS = 0.032) was 7.6 °C lower than that of NC, and this value was consistent with the previous report [38] and the results of viscosity temperature curve analysis. Gelation occurs when hot solutions are cooled and involves coil–helix transition, followed by helix aggregation [39]. The coil to double helix transition is the key step for gelation. The introduction of the large MAH group may weaken the hydrogen bond and prevent the formation of a double helix structure. The κCar double helix broke prematurely during heating because of the weak bonds and intermolecular forces; thus, MC began to melt at a low temperature. The low melting temperature was ascribed to the low energy required to break down the network, indicating that the gels of MC were less stable with loose aggregation than those of NC [40,41]. The decrease in dissolving, gelling, and melting temperatures allows for the joint application of temperature-sensitive substances and κCar.

### 2.4. Characterization and Properties of NC/MC Films

#### 2.4.1. Morphology

**Figure 3:**
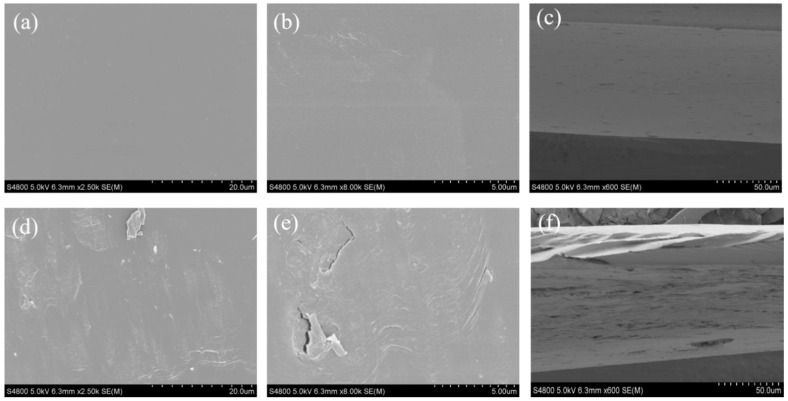
(**a**,**b**) SEM surface images of NC films magnified by 2500× and 8000×, respectively; (**c**) SEM cross-section image of NC films; (**d**,**e**) SEM surface images of MC films magnified by 2500× and 8000×, respectively; (**f**) SEM cross-section image of MC films.

To investigate the microstructure of the NC and MC films, the morphology of the surface and cross-section of the films was observed, as shown in Figure 3. The NC film showed a smooth and compact surface (Figure 3a,b), but the MC film exhibited an obvious rough surface (Figure 3d,e). The results indicated that the surface roughness of κCar-based films increased after modification with MAH. Moreover, cross-sectional SEM micrographs of NC and MC films are shown in Figure 3c,f, respectively. The cross-section images confirmed that the cross-section of the κCar-based film changed from dense to porous after modification. All changes in the microstructure revealed that the modification reaction had taken place. Similar microstructural results have been reported for OSA-modified sweet potato starch film [42].

#### 2.4.2. Optical Properties

The color parameters are integral to the appearance and consumer acceptance of packaged products [43]. The color of the NC and MC films was evaluated by using *L* (whiteness and blackness), *a* (redness and greenness), and *b* (yellowness and blueness) values and *∆E*. As shown in Table 2, the NC and MC films presented negative values of *a* and positive values of *b*, signifying their greenish yellow color. Compared with NC films, the MC films leaned toward yellow, as suggested by their higher *b* values. Moreover, the values of *a* and *∆E* did not significantly differ between the NC and MC films. The *L* value of MC films was comparative to that of NC films, implying that both have high transparency. These results could be attributed to the high transparency of NC and MC, which brightened the films.

**Figure 4:**
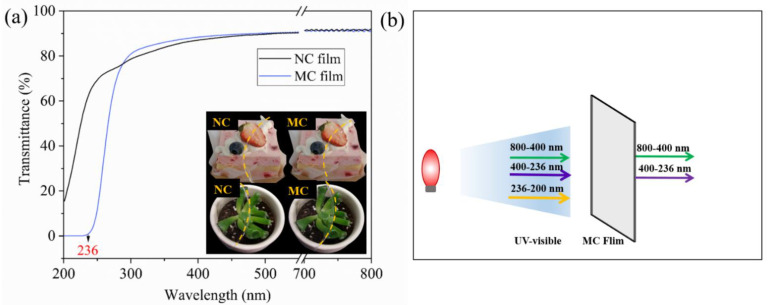
(**a**) Light transmittance of the films and (**b**) UV absorbability of the MC film.

Transmittance strongly affects film functionality due to its great effect on the appearance of packaged products [44]. Figure 4a shows the transmittance curves of the films. The transmittance of NC and MC films was high at above 80% in the visible light range of 400–800 nm. However, in the UV region of 200–400 nm, the transmittance of both films decreased to different degrees with the reduction in wavelength. Within the wavelength of 236–300 nm, the transmittance of the NC films decreased from 78% to 62%, and that of the MC films decreased drastically from 81% to 0%. These results showed that the MC films could completely absorb UV light in the range of 200–236 nm. This phenomenon could be attributed to the introduction of MAH groups, resulting in conjugated double bonds in the MC film. Lu et al. [45] found that the neat polyvinyl alcohol film has an extremely high transmittance in the whole UV–Vis light range. After disassembled polydopamine (PDA) was introduced into the film, the transmittance in UV and visible regions decreased seriously due to the conjugation effect of PDA. 

#### 2.4.3. Mechanical and Water Affinity Properties

**Figure 5:**
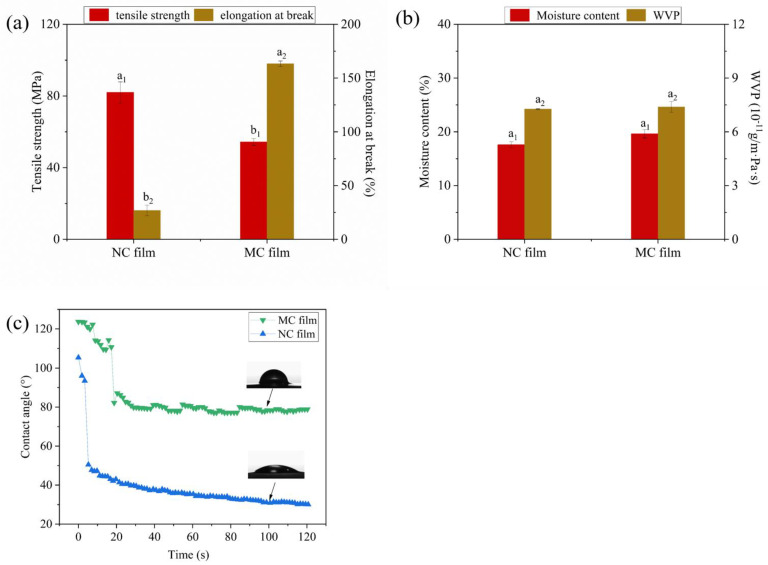
(**a**) Tensile strength and elongation at break; (**b**) moisture content and water vapor permeability; (**c**) contact angle curves along with time of the films. a_1_, b_1_ indicate significant differences in tensile strength in Figure 5a (*p* < 0.05); a_2_, b_2_ indicate significant differences in elongation at break in Figure 5a (*p* < 0.05); a_1_ indicates no significant differences in moisture content in Figure 5b (*p* > 0.05); a_2_ indicates no significant differences in water vapor permeability in Figure 5b (*p* > 0.05).

The mechanical properties of films include tensile strength (TS) and elongation at break (EB), with the former elaborating the resistance of the film to elongation and the latter denoting the ductility of the film. Figure 5a shows the TS and EB of the NC and MC films. Compared with that of the NC films, the TS of the MC films decreased from 82.0 MPa to 54.3 MPa, and the EB increased from 26.9% to 163%. The decrease in TS in the MC films may be due to the substitution of hydroxyl groups in the chain of κCar, resulting in the reduction of intermolecular hydrogen bonds and the weakening of the polymerization ability of κCar, which destroyed the integrity of the MC films. Li et al. [42] confirmed that the presence of octenyl groups weakened the molecular interactions within the film matrix and thus reduced the TS of octenyl succinated sweet potato starch film. Meanwhile, the reduction of intermolecular forces increased the mobility and deformation of carrageenan molecular chains, which in turn enhanced its flexibility and ductility and thereby increased the EB.

Moisture content, water vapor permeability (WVP), and water contact angle were used to determine the water resistance of the films. The effects of κCar modification on the water resistance of MC films are shown in Figure 5b. No significant differences (*p* > 0.05) in moisture content and WVP were noticed between the NC and MC films, indicating that κCar modification only had a slight effect on the moisture content and WVP of κCar films. The water contact angle reflects the hydrophilic/hydrophobic surface properties of the materials. In general, a material with hydrophobicity also has a high contact angle. As can be seen in Figure 5c, the contact angles of both MC films were higher than those of NC films as time increased. The contact angles of MC and NC films were kept at approximately 78° and 30°, respectively. This revealed that the surface hydrophobicity of κCar-based films was improved after MAH modification, which could be related to the increased roughness of the MC films compared with that of the NC film, as shown in the SEM micrographs (Figure 3). In addition, the introduction of hydrophobic MAH molecules and the reduction of hydrophilic groups (-OH) due to the covalent bonds formed between MAH molecules and κCar were other reasons for the increased surface hydrophobicity of MC films [46,47]. 

## 3. Materials and Methods

### 3.1. Materials 

Food-grade κCar (gel strength ≥ 1300 g/cm^2^, ash content 15–25%, and moisture content ≤ 12%) was obtained from Greenfresh (Fujian) Food Stuff Co., Ltd. (Fujian, China). MAH was purchased from Vertellus (Shanghai, China) Chemical Co., Ltd. (Shanghai, China). Sodium hydroxide, hydrochloric acid, anhydrous ethanol, isopropanol, glycerol, and sodium bromide were all of analytical grade and purchased from Sinopharm Chemical Reagent Co., Ltd. (Shanghai, China). All chemicals were used as received without further purification.

### 3.2. Synthesis of MC 

MC was prepared using MAH according to a previous report with minor modifications [48]. Initially, different concentrations of κCar powder based on the volume of the reaction system (*w*/*v*) were dispersed in 200 mL of ethanol solution (80%) and magnetically stirred at different temperatures. Then, MAH dissolved in absolute alcohol with a ratio of 1:3 (MAH: absolute alcohol, *w*/*v*) was added dropwise to the previous mixture for 1 h and then continuously stirred for 0.5–2.5 h. pH of the suspension was adjusted (7.0–9.5) by adding the alkaline solution (1 M NaOH). At the end of the reaction, the obtained mixture was washed three times with 80% ethanol and oven-dried overnight at 55 °C.

### 3.3. Determination of DS 

The DS of MC was measured by the method described earlier [49]. In brief, 1.0 g of sample was accurately weighed and soaked with 5 mL of isopropanol solution. The suspension was stirred for 30 min in 15 mL of 2.5 mol/L hydrochloric acid–isopropanol solution. The mixture was added with 30 mL of 90% isopropanol solution and then stirred for another 10 min. The residue was washed with 60% ethanol until no Cl^−^ remained (as examined with silver nitrate solution), then dispersed in 100 mL of deionized water, and dissolved in a microwave oven for 3 min. The solution was titrated with 0.02 M sodium hydroxide using phenolphthalein as an indicator. Consumption volume was recorded. DS was calculated with the following equations:

(1) DS = (0.385 × A)/(1 − 0.098 × A)

(2) A=(C × V)/W

where C is the concentration of NaOH (mol/L), V refers to the consumed volume of NaOH (mL), and W represents the quality of the sample (g). The molecular weight of the disaccharide unit is 385, and that of MAH group is 98.

### 3.4. Characterization of MC

#### 3.4.1. FT-IR

The FT-IR spectrum of κCar power was determined on a Nicolet iS50 FT-IR spectrometer (Thermo Fisher Nicolet, Waltham, MA, USA) at room temperature. Prior to pellet preparation, the samples were dried at 55 °C for 12 h to remove any moisture, finely ground with KBr in a ratio of 1:100 (*w*/*w*) to generate a thin pellet, and scanned within the wavenumber range of 400–4000 cm^−1^. 

#### 3.4.2. TGA

TGA of κCar power was conducted with a thermogravimetric analyzer (TA, Q500, New Castle, DE, USA) between 30 °C and 600 °C at a heating rate of 10 °C/min in a N_2_ atmosphere.

### 3.5. Determination of Physical Properties

Gel strength was determined using the method described by Lee et al. [50]. In brief, 100 mL of hot κCar solution dissolved in 1.5% (*w*/*v*) water containing 0.2 g of KCl was poured into a Petri dish with 30 mm diameter and 21–22 mm depth and then allowed to gel at room temperature for 15 h. Gel strength was measured by the load (g/cm^2^) causing a cylindrical plunger (1 cm^2^ cross-section) to suddenly break the gel. 

The viscosity of κCar solution at 75 °C was determined using a Brookfield DV-C Viscometer operating at 50 rpm with spindle #62. (Brookfield, Middleboro, MA, USA). In brief, 1.5% (*w*/*v*) κCar solution (in distilled water) was prepared by boiling in a microwave oven for 8 min. The hot solution was then placed in a 75 °C water bath pot insulated for 30 min [51]. Apparent viscosity was measured using a Brookfield Synchrolectric Viscometer (USA) as described by Zhao et al. [52].

Another 1.5% κCar solution (30 mL) was prepared and poured into a 50 mL centrifuge tube to determine the water holding capacity. The gel was placed in a freezer for 2 h and frozen for 8 h before defrosting. Pressure dehydration with weight was conducted, and the water was poured out. Volume and dehydration rate were calculated (V/30 × 100).

Transparency was measured by transmittance (%) at 700 nm with distilled water as blank. The sample solution (1.5%, *w*/*v*) was placed in the colorimetric ware and then incubated at room temperature for 12 h [53]. 

Whiteness was determined using a colorimeter in ADC1-WS1.

The gelling and gel melting temperature of κCar were measured at shear deformation by using the TA instrument TRIOS rheometer and the modified method of Derkach et al. [54]. Temperature scanning was performed from 20 °C to 80 °C, followed by 80 °C to 20 °C at a scan rate of 2 °C/min, an amplitude of deformation *γ* = 1%, and a frequency *ω* = 6.28 c^−1^. The diameter of the cone was 40 mm, the angle between the cone and plate was 1 grad, and the gap between the cone top and the plate was 0.1 mm.

Dissolving temperature was measured by preparing a 10 mL solution of κCar glue (1.0%) in a test tube in a water bath, and the temperature was increased from 60 °C to 100 °C at 1 °C/10 min. The dissolving temperature was recorded when the κCar powder was completely dissolved [55].

### 3.6. Determination of Chemical Compositions

The sulfate content of κCar was assessed by the barium chloride gelatin method with K_2_SO_4_ as the standard [51], and 3,6-AG content was determined colorimetrically using the resorcinol–acetal method of Freile-Pelegrín and Robledo [56].

### 3.7. Preparation of NC/MC Films

Solution casting was used to prepare the films [57]. In brief, 3.0 g of NC/MC powder was placed in a beaker and added with 150 mL of distilled water to reach the mass fraction 2%. The beaker was placed on a magnetic stirrer at 80 °C and stirred for 30 min to complete dissolution. The carrageenan solution was added with 0.9 g of glycerin (30 wt% of carrageenan), magnetically stirred at 80 °C for 30 min to evenly mix the evaporated water with hot water, and sealed with plastic wrap. The beaker was placed in a constant temperature water bath at 75 °C for 1 h to stabilize the carrageenan solution and eliminate bubbles. Afterward, 10.0 g of the mixed solution was isolated, poured into a glass Petri dish (90 mm in diameter), and cooled down naturally at room temperature to form a gel. The gel was placed in a 55 °C blast drying oven for 24 h. The dried films were peeled off from the glass Petri dishes and conditioned in a humidity chamber controlled at 25 °C and 50% relative humidity (RH) for at least 2 days before further analysis.

### 3.8. Surface Morphology and Cross-Section of NC and MC Films

The microstructures of surface and cross-section (cryofractured) of NC and MC films were obtained using scanning electron microscopy (S-4800, Hitachi Co., Tokyo, Japan) with an acceleration voltage of 5 kV. All of the samples were coated with platinum using a vacuum sputter coater before measurement.

### 3.9. Performance Measurement for NC and MC Films

#### 3.9.1. Thickness Measurement

A micrometer (Thickness Gauge; Mitutoyo Co., Tokyo, Japan) was used to randomly select 10 points (including the center point) on the film to be tested to measure its thickness, and the average value was taken as the film thickness.

#### 3.9.2. Determination of Optical Properties

The surface color of NC and MC films was evaluated using a colorimeter (WSC-S; Shanghai Precision & Scientific Instrument Co., China). A white color plate (L* = 91.86, a* = −0.88, b* = 1.42) was employed as a standard background color. Color parameters such as Hunter *L*, *a*, and *b* (*L*: lightness, *a*: greenness–redness, and *b*: blueness–yellowness) values were determined by measuring at the five random points of each film sample. The total color difference (*∆E*) of the film was calculated as follows:

(3) $$\Delta E=\sqrt{{(L-L^{*})}^{2}+(a-a^{*}{)}^{2}+(b-b^{*}{)}^{2}}$$

where *L*, *a*, and *b* are the color values of the NC and MC films, respectively.

Film light transmission was measured by transmittance (%) with the aid of a UV–visible spectrophotometer (Cary-60, Agilent, Santa Clara, CA, USA) operating in the wavelength range of 200–800 nm with an accuracy of 1 nm. Three replicates of each film were tested.

#### 3.9.3. Determination of Mechanical Properties

Rectangles of 45 × 20 mm were cut from the film, and the mechanical properties of NC/MC films were measured by a texture analyzer (TMS-PRO, Food Technology Co., Sterling, VA, USA). The test speed was 1 mm/s, and the initial clamping distance was 30 mm [58]. Six parallels were measured for each sample, and the average value was taken. The mechanical properties of carrageenan films were calculated according to the following formulas:

(4) $$\mathrm{TS}\;=\;F_{\max}/S$$

where TS is tensile strength, MPa; *F_max_* is the maximum load when the film is broken, N; and *S* is the cross-sectional area of membrane, mm^2^.

(5) $$\mathrm{EB}=(l\;-l_{0})/l_{0}\;\times \;100$$

where EB is the elongation at break, %; *l* is the length of the film after stretching, mm; and *l_0_* is the initial length of the film, mm.

#### 3.9.4. Determination of Water Affinity Properties

Water contact angle was determined using a contact angle meter (CA-100C; Shanghai Innuo precision instruments Co., Shanghai, China). For each measurement, 5 µL of deionized water was dropped onto film surface. Contact angle dynamics with time was measured over 2 min and plotted as curves.

Film moisture content was analyzed by gravimetry in triplicate by drying the samples at 105 °C for 24 h [59].

Water vapor permeability was determined following the standard method of ASTM E96-95 with modification [60]. Film samples were tightened on the top of weighing cups containing 30.00 g of silica gel. These weighing cups were stored in a specific environment set at 25 °C and 50% RH. Weight loss from each cup was measured every 12 h for 7 days. WVP (g/m·s·Pa) was determined from the slope of the plot of weight change of the cup versus time and calculated as follows:

(6) WVP=(Δm × X)/(S × Δt × Δp)

where *∆m* (g) represents the weight change of the weighing cup after 7 days; *X* (m) and *S* (m^2^) are the thickness and area of the film, respectively; *∆t* (s) denotes the time intervals; and *∆p* (Pa) is the partial water vapor pressure difference across the two sides of the film.

### 3.10. Statistical Analysis

All experimental data were processed by using SPSS (v18.0; SPSS Inc., Chicago, IL, USA). Differences among the average values were obtained via q test or paired-samples *t* test (*p* < 0.05).

## 4. Conclusions

κCar was successfully modified by MAH. The maximum DS and minimum gel strength were reached under the following optimal reaction conditions: 30 °C reaction temperature, 3.5 h reaction time, 8% MAH concentration, 7.5% κCar concentration, and pH 8–8.5. FT-IR spectroscopy showed that characteristic absorptions in MC occurred on the ester carbonyl groups at 1734 cm^−1^ and the carboxylate at 1576 cm^−1^. TGA results revealed that the thermal stability of MC decreased. The physicochemical properties of MC including gelling temperature, gel strength, viscosity, and 3,6-AG content were all decreased, whereas its water holding capacity and transparency were remarkably improved. The increase in MC transparency (from 81.9% to 91.6%) favored the color and transparency of MC films. The SEM indicated that the surface of the MC films had become rougher after modification. Moreover, the introduction of MAH groups into κCar through modification endowed the film with enhanced hydrophobicity and ability to absorb UV light in the range of 200–236 nm. The reduced intermolecular forces of κCar also led to a substantial increase in EB from 26.9% to 163%. Therefore, the novel MC with MAH could be effectively exploited in polysaccharide-based films.

## Figures and Tables

**Table 1:** Other physicochemical properties of κCar.

Physicochemical Properties	NC	CC	MC/DS = 0.015	MC/DS = 0.032
Gel strength (g/cm^2^)	1441 ± 20 ^a^	1432 ± 19 ^a^	1329 ± 17 ^b^	759 ± 23 ^c^
3,6-AG content (%)	22.5 ± 0.3 ^a^	22.4 ± 0.1 ^a^	18.8 ± 0.1 ^b^	17.1 ± 1.3 ^b^
Sulfate content (%)	25.7 ± 1.7 ^a^	23.2 ± 3.6 ^a^	22.5 ± 1.7 ^a^	22.1 ± 1.1 ^a^
Transparency (%)	81.9 ± 0.1 ^d^	86.9 ± 0.1 ^c^	87.6 ± 0.0 ^b^	91.6 ± 0.1 ^a^
Whiteness (%)	57.3 ± 0.2 ^b^	54.3 ± 0.2 ^d^	58.5 ± 0.2 ^a^	56.9 ± 0.1 ^c^
Dissolving temperature (°C)	96.6 ± 0.1 ^a^	95.1 ± 2.6 ^a^	93.1 ± 2.6 ^a^	93.1 ± 0.1 ^a^
Gelling temperature (°C)	40.9 ± 0.2 ^a^	39.4 ± 0.9 ^b^	35.8 ± 0.4 ^c^	33.3 ± 0.4 ^d^
Melting temperature (°C)	55.7 ± 0.1 ^a^	54.6 ± 0.4 ^b^	54.0 ± 0.9 ^b^	50.4 ± 0.0 ^c^

Values are mean ± standard deviation. Different lowercase superscripts within the same row indicate significant differences (*p* < 0.05). CC: control κCar.

**Table 2:** Color parameters of the films.

	*L*	*a*	*b*	*∆E*
NC film	89.29 ± 0.22 ^a^	−0.92 ± 0.06 ^a^	2.74 ± 0.08 ^b^	2.89 ± 0.20 ^a^
MC film	89.51 ± 0.25 ^a^	−1.04 ± 0.05 ^a^	3.18 ± 0.02 ^a^	2.94 ± 0.21 ^a^

Values are mean ± standard deviation. Different lowercase superscripts within the same column indicate significant differences (*p* < 0.05).

## Data Availability

Data are contained within the article.
